# The association between circulating leukocytes and inflammatory bowel disease: a two-sample Mendelian randomization study

**DOI:** 10.3389/fmed.2024.1399658

**Published:** 2024-05-27

**Authors:** Li Tian, Xiaobin Yang, Yansen Zheng, Chaosheng Peng

**Affiliations:** ^1^Day Diagnosis and Treatment Department, The Sixth Medical Center of PLA General Hospital, Beijing, China; ^2^Medical School, Huanghe Science and Technology College, Zhengzhou, China

**Keywords:** inflammatory bowel disease, ulcerative colitis, Crohn’s disease, circulating leukocytes, Mendelian randomization

## Abstract

**Background:**

Inflammatory bowel disease (IBD) is a highly prevalent, recurrent, chronic intestinal inflammatory disease. Several observational studies have shown that circulating leukocytes are strongly associated with IBD. However, whether alterations in leukocytes are causally related to IBD remains uncertain. The present study explores this issue with the Mendelian randomization (MR) analysis method.

**Methods:**

The Genome wide association study (GWAS) statistical data related to circulating leukocytes and IBD were obtained from the Blood Cell Consortium and the IEU Qpen GWAS project, respectively. Inverse variance weighting (IVW) was used as the main MR analytical method, coupled with a series of sensitivity analyses to ensure the reliability of the results.

**Results:**

The results of IVW showed that increased monocyte count (especially CD14- CD16+ monocyte absolute counts) was negatively correlated with the risk of IBD and its main subtypes. Increased neutrophil count was positively associated with the risk of IBD and ulcerative colitis. Meanwhile, there was no causal relationship between basophil, eosinophil, lymphocyte counts and IBD risk.

**Conclusion:**

These results indicate that a causal relationship exists between circulating leukocytes and the risk of IBD and its subtypes, which confirms the important role that the leukocyte immune system plays in IBD. Our findings provide additional research directions for the clinical prevention and treatment of IBD.

## 1 Introduction

Inflammatory bowel disease (IBD) is an idiopathic and inflammatory chronic intestinal disease, characterized by typical clinical symptoms such as diarrhea, abdominal pain, and bloody stools (1, 2). IBD significantly affects inflicted people’s daily life, mental state, and work efficiency (3, 4). In the past decades, tremendous progress has been made in basic research and clinical treatment of IBD, but the current clinical treatment and management still face significant challenges due to its complex pathogenesis and individual specificity (5, 6). Therefore, elucidating the pathogenesis of IBD may provide new strategies for clinical treatment approaches.

IBD includes Crohn’s disease (CD) and ulcerative colitis (UC), and current research indicates that IBD is closely associated with environmental factors, genetic susceptibility, and the intestinal mucosal immune system (7, 8). The adaptive immune response has classically been considered to play a major role in the pathogenesis of IBD (9, 10). Leukocytes are complex and important players in the intestinal immune system, which includes granulocytes (neutrophils, eosinophils, and basophils), monocytes, and lymphocytes (11). Neutrophil-derived cytokines and chemokines enhance local immune responses in the intestinal mucosa and mediate impaired epithelial barrier function and tissue damage (12). Additionally, highly activated neutrophils release neutrophil extracellular traps (NETs) to trap microorganisms and prevent transmission, and NETs were found to accumulate and maintain inflammatory signals in the inflamed intestinal mucosa of patients with active UC (13, 14). Eosinophils are also increased in the intestinal mucosa of IBD patients and when activated, lead to degranulation and release of granule proteins such as major basic proteins and cytotoxic eosinophil cationic protein (ECP). Activated eosinophils secrete different cytokines to activate other immune cells such as mast cells, which are thought to cause tissue inflammation and damage (15).

Circulating leukocytes are key parameters for evaluating immune cell homeostasis. So far, several observational studies have linked circulating leukocytes to the onset of IBD (16–18). An observational cohort study correlates monocyte counts with recurrent treatment in patients with IBD. After 16 months of continuous monitoring of 95 IBD patients, the results showed that there is a significant difference between relapse and remission in monocyte counts, and monocytes and fecal calprotectin were associated with relapses according to multivariate analysis (19). Another study categorized patients with active or inactive UC and CD according to the severity of the disease by recording neutrophil and lymphocyte counts and calculating their ratios. The results showed that the neutrophil-to-lymphocyte ratio (NLR) in IBD subjects was strongly associated with active disease (20). However, these observational studies have not clarified the role of leukocyte counts in the pathogenesis of IBD and have not been able to rule out the influence of confounding factors and reverse causation. Therefore, better research methods are needed to elucidate the role of leukocyte counts in the pathogenesis of IBD.

Mendelian randomization (MR) analysis is a method that utilizes genetic data to evaluate the causal effects caused by modifiable non-genetic exposure factors. For example, randomized controlled studies on the effect of diet on IBD. Dietary intake alone may influence the risk of IBD. Or the intake of certain foods may alter the risk of IBD through effects on the host immune system, gut barrier, and gut microbiota. Conversely, it may be that IBD alters a patient’s dietary habits (21, 22). The MR analysis method minimizes confounding factors, measurement error, and reverse causality that may affect traditional randomized controlled trials. Since genetic variants are randomly assigned during meiosis, genotype distribution precedes acquired exposure in time (23–25). In this study, the causal relationship between circulating leukocytes and IBD was explored using MR analysis. Based on our study of the pathogenesis of IBD from the aspect of genetics, we aim to provide some theoretical basis for the new direction of clinical treatment research of IBD.

## 2 Materials and methods

### 2.1 Data sources for circulating leukocytes and IBD

The summary statistics relating to circulating leukocytes were obtained from the largest meta-analyzed genome-wide association studies (GWAS) data provided by the Blood Cell Consortium (26). The sample size was 563946 patients. The aggregated data from the GWAS studies for monocyte subtypes was obtained from a cohort study on genetic variations in immune cells among indigenous Sardinians, which included a sample size of 3,757 individuals (27). For the summary data related to IBD and its main subtypes, we used FinnGen research project data from the Finnish Biobank. The GWAS data for IBD included 5,673 cases and 213119 control subjects; GWAS for UC, included 4,320 cases and 210300 control subjects; and GWAS for CD, included 807 cases and 210300 control subjects. The data used in this study were obtained from existing publications or public databases. Ethical approval and informed consent were obtained for these publications or databases and therefore no additional ethical approval was required. Detailed information on the race profile and GWAS-related to the study characteristics are summarized in Supplementary Tables 1–3.

### 2.2 Genetic instrument selection criteria

In our MR analysis, single nucleotide polymorphisms (SNPs) related to circulating leukocytes and IBD were used as instrumental variables (IVs). The screening criteria for IVs were as follows: SNPs that are significantly (*p* < 5 × 10^–8^) correlated with circulating leukocytes were considered as IVs. Due to the small number of SNPs in the actual screening process, the threshold for evaluating significance level is expanded to *p* < 5 × 10^–6^; PLINK clumping was used to avoid bias caused by linkage imbalance between SNPs, the screening conditions for SNPs need to meet *r*^2^ < 0.01 and kb = 5000; and the F-statistic is greater than 10, indicating a strong correlation between SNPs and the exposure, which can avoid the impact of weak IVs bias.

### 2.3 Statistical analysis

In this study, the causal relationship between circulating leukocytes and IBD, UC, and CD was assessed by several methods, including inverse variance weighting (IVW), MR-Egger, weighted median, MR-Raps, MR-Presso, and Radial MR. Each MR method can infer different results, and when the results are consistent, it is more indicative of the reliability of the results. Fixed-effects IVW is the most effective test in two-sample MR analysis and is the primary method in this study. MR-Egger assumes all IVs are invalid and have relatively low statistical efficacy, but can provide correction for multiple effects. The weighted median method assumes 50% of IVs are invalid and can provide accurate causal estimates. Similarly, several methods were also used in this study for sensitivity analysis. MR-Egger was used to detect horizontal pleiotropy of IVs, with intercepts and *p*-values reflecting effect sizes and biases. IVW and MR-Egger were used to identify heterogeneity of IVs and quantified with Cochran’s Q statistical test, where a *p*-value of less than 0.05 indicated the presence of heterogeneity. The *p*-value is then corrected by MR-Presso to exclude outliers, and random-effects IVW modeling. MR analyses were performed using the Two Sample MR package in R software (version 4.2.0).

## 3 Results

**FIGURE 1:**
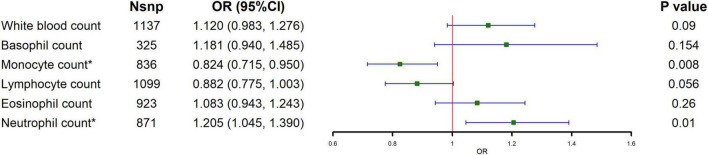
Causal estimates given as odds ratios (ORs) and 95% confidence intervals (CIs) for the effect of circulating leukocytes on IBD.

**FIGURE 2:**
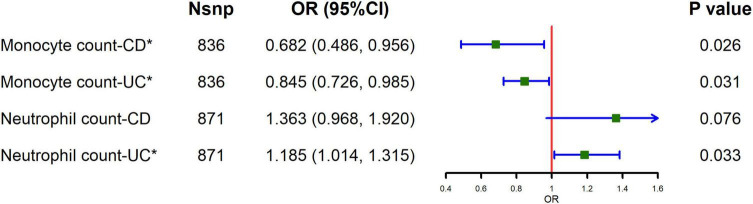
Causal estimates given as odds ratios (ORs) and 95% confidence intervals (CIs) for the effect of monocytes and neutrophils on CD and UC.

The causal relationship between circulating leukocytes and IBD is shown in Figure 1 and Supplementary Table 4. The results of our MR analysis using IVW showed that increased monocyte count was negatively correlated with the risk of IBD [odds ratio (OR) = 0.824, 95% confidence interval (CI) = 0.715–0.95, *p* = 0.008], and increased neutrophil count was positively associated with IBD risk (OR = 1.205, 95% CI = 1.045–1.39, *p* = 0.010), and there was no causal relationship between basophil, eosinophil and lymphocyte counts and IBD risk. Meanwhile, we examined the relationship between monocyte count and neutrophils count for the risk of the major subtypes of IBD. The results showed that monocyte count was negatively associated with the risk of both UC and CD, as shown in Figure 2 and Supplementary Table 5 (CD: OR = 0.682, 95% CI = 0.486–0.956, *p* = 0.026; UC: OR = 0.845, 95% CI = 0.726–0.985, *p* = 0.031). Meanwhile, neutrophil count was positively associated with the risk of UC only (OR = 1.185, 95% CI = 1.014–1.315, *p* = 0.033). The results of IVW were also supported by other MR analysis methods. There was no evidence of horizontal pleiotropy based on the MR-Egger intercept test, and Cochran’s Q test detected significant heterogeneity in some cell types, and a random-effects IVW model was used to estimate MR effect sizes, with results consistent with previous ones (Supplementary Table 7).

**FIGURE 3:**
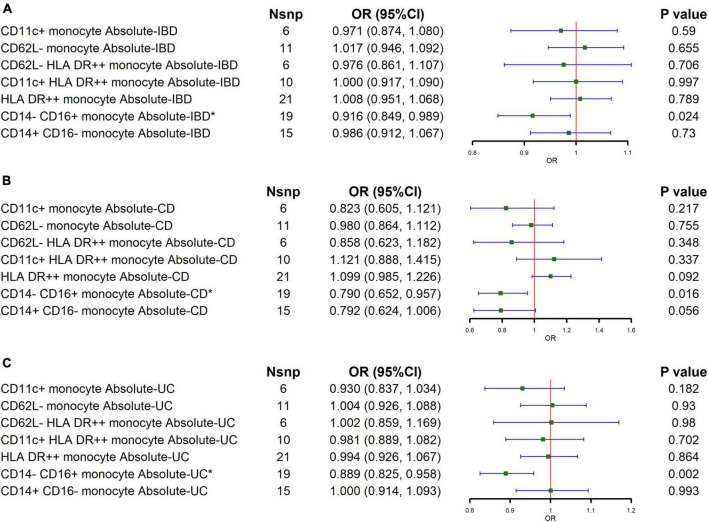
Causal estimates given as odds ratios (ORs) and 95% confidence intervals (CIs) for the effect of the major subtypes of monocytes on IBD and its main subtypes. **(A–C)** The effects of major monocyte subtypes on IBD, CD, and UC, respectively.

To explore the major subtypes of monocytes that reduce the risk of IBD, we performed MR analysis for the absolute counts of different monocyte subtypes. The results of IVW showed a negative correlation between the CD14- CD16+ monocyte absolute counts and the risk of both IBD and its major subtypes (IBD: OR = 0.916, 95% CI = 0.849–0.989, *p* = 0.024; CD: OR = 0.790, 95% CI = 0.849–0.989, *p* = 0.016; UC: OR = 0.889, 95% CI = 0.849–0.989, *p* = 0.002), as shown in Figure 3 and Supplementary Table 6. MR-Egger regression analyses showed no potential for pleiotropy. The Cochran’s Q test showed that certain monocyte subtypes had significant heterogeneity, so a random effects model was used to estimate the MR effect (Supplementary Table 7).

## 4 Discussion

This study is aimed to define whether the connection between circulating leukocytes and IBD is causal. The current MR analyses in our study suggested that genetically determined high neutrophil count may increase the risk of IBD and UC, and monocyte count (especially CD14- CD16+ monocyte absolute count) may reduce the risk of IBD and its main subtypes. In addition, there was no evidence from MR analyses indicating that other types of leukocytes, as well as monocyte subtypes, increased or decreased the risk of IBD.

Neutrophils are the most numerous leukocytes in the body’s immune cells, which can rapidly accumulate at the location of infection or tissue injury and are the first line of defense in the innate immune response (28). A current research has suggested that the role of neutrophils in the etiopathogenesis of IBD is like a double-edged sword. On the one hand, neutrophils recognize, phagocytose, and kill pathogens through reactive oxygen species (ROS). Neutrophils with antimicrobial potential are produced by respiratory bursts, and also release NETs to eliminate microorganisms and prevent the spread of pathogens (29). On the other hand, neutrophils produce or release ROS, several proteases, and pro-inflammatory cytokines such as Interleukin 8 (IL-8), Tumour Necrosis Factor alpha (TNF-α), and leukotriene B4. These cytokines disrupt the intestinal epithelial barrier, absorb monocytes and additional neutrophils, and activate redox inflammatory responses that lead to tissue damage (30, 31). The results of our MR analysis support the latter, of which immune responses actually have an inversely negative effect. Our study confirmed that genetically increased neutrophils are more likely to increase the risk of IBD, combined with other negative effects of the cytokines.

Several studies have explored the relationship between monocyte count and IBD. AS Mee et al. demonstrated that the peripheral blood monocyte counts were elevated in patients with IBD and correlated strongly with disease activity (32). Another study retrospectively analyzed 727 pediatric patients diagnosed with IBD and investigated whether monocyte counts could serve as a predictor of eventual relapse in patients who relapsed after discontinuation of the drug (18). The results of this study showed that patients with high monocyte counts experienced relapse after discontinuation of biologic agents. On the contrary, our MR analysis showed that genetically increased monocytes, and a count of non-classical monocytes (CD14- CD16+ monocyte), may be associated with a lower risk of IBD. The possible mechanism of action for nonclassical monocytes is associated with adhesion, production of complement components, and Fcγ-mediated phagocytosis. During the development of IBD, the intestinal epithelial barrier is compromised, allowing microbes to enter the tissue (33). Non-classical monocytes are recruited to the gut via α4β7 integrin, which transforms into macrophages and promotes wound healing (34, 35). The application of integrin antibodies against non-classical monocytes also confirmed the mechanism. The anti-α4β7 integrin antibody, vedolizumab, prevents the supplement and further differentiation of non-classical monocytes into wound healing promoting macrophages from entering the intestine and prolonging wound healing time (35).

Previous randomized controlled trials have reported that IBD is associated with high levels of lymphocyte counts, eosinophil counts, and basophil counts (36–38). However, there has been no evidence to support the causal connection between genetically predicted lymphocyte count, eosinophil count, and basophil count with IBD. The lack of association may be due to the influence of confounding factors in observational findings. Compared with healthy controls, biopsies of the intestinal mucosa of patients with IBD showed mucosal eosinophilia. Eosinophils secrete eosinophil granule proteins (EGPs), such as ECP, EPO, EDN, and MBP, and these products are found at elevated levels in tissues and fecal effluents, providing indirect support for the development of IBD (39). Lymphocytes, as a type of circulating white blood cells, have specific phenotypes and functions in different subtypes. Therefore, the total number of lymphocytes cannot fully represent the heterogeneity of lymphocyte subpopulations (40).

This study has proved the causal relationship between circulating leukocytes and IBD through two-sample MR analysis. Our GWAS-based MR study avoids costly and time-consuming randomized controlled studies, and the bias of results caused by confounding factors such as environment, society, behavior, and psychology. At the same time, causality analysis by genetic variation of circulating leukocytes and IBD has higher reliability and less measurement error. Despite the valuable approaches of our study, there are still some unavoidable problems in this research. First, differences in gene frequency and disease incidence can only be analyzed for one species, and the analysis of mixed populations will make population stratification a new confounding factor. Therefore, European populations with larger sample sizes and more complete GWAS data were selected for analysis in this study. Secondly, the participants in this study were of exclusively European ancestry, and there was no evidence that the results of the MR analyses differed significantly across populations of varying ethnic groups, so further data collection and analysis is needed to determine whether the findings are applicable to other populations. In addition, the MR method mainly focuses on data analysis to assess causal effects statistically, and cannot provide a reasonable biological explanation for causality, which needs to be continuously explored and verified in subsequent basic research and clinical trials.

## 5 Conclusion

Currently, very few studies that have investigated the causal relationship between circulating leukocytes and IBD and its major subtypes using MR methods. This study provides evidence that higher neutrophil counts may increase the risk of IBD and UC. In addition, we found that higher monocyte cell counts, especially non-classical monocytes, may reduce the risk of IBD and its main subtypes. Our findings shed more light on the role of systemic immune changes in the pathogenesis of IBD and provide support for the prevention as well as clinical treatment of IBD.

## Data availability statement

The original contributions presented in the study are are included in this article/Supplementary material. Data on IBD were downloaded from https://gwas.mrcieu.ac.uk/. The summary statistics relating to circulating leukocytes were obtained from the Blood Cell Consortium. Further inquiries can be directed to the corresponding author.

## Author contributions

LT: Writing – review and editing, Writing – original draft, Conceptualization. XY: Writing – review and editing, Writing – original draft, Data curation. YZ: Writing – review and editing, Writing – original draft, Validation, Investigation. CP: Writing – review and editing, Writing – original draft, Supervision, Methodology, Conceptualization.
